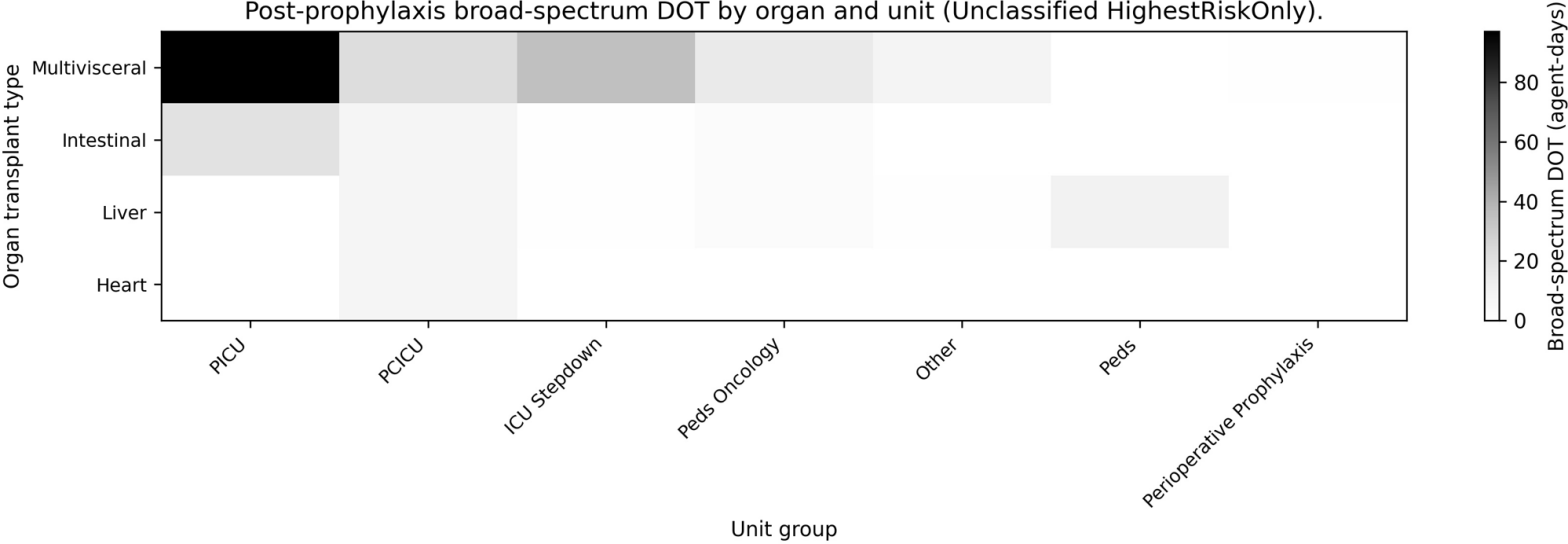# 144 Clinical Decision Support Systems for Infection Prevention and Control: A Survey of Adoption, Maturity, and Barriers to Implementation

**DOI:** 10.1017/ash.2026.10437

**Published:** 2026-06-23

**Authors:** Lakshmy Arakoni, Kaitlyn Rivard, Blanca Gonzalez, Jessica Alban, Venkatraman Arakoni, Charles Foster

**Affiliations:** 1 University of Toledo; 2 Cleveland Clinic; 3 Cleveland Clinic Foundation; 4 Cleveland Clinic Children’s

## Abstract

**Background:** Pediatric solid organ transplant recipients receive broad-spectrum antibiotics. The CDC NHSN Antimicrobial Use and Resistance (AUR) Module reports days of therapy (DOT) by pediatric Standardized Antimicrobial Administration Ratio (SAAR) categories, but those unit-level summaries do not identify the contexts that drive broad-spectrum DOT. We used a procedure-anchored framework to quantify post-prophylaxis broad-spectrum exposure and attribute it to blood-culture evaluation, surgical site infection (SSI), and subsequent procedures. **Methods:** We analyzed 164 pediatric transplant procedures (heart 59, liver 58, kidney 29, intestinal 8, multivisceral 10) with follow-up through postoperative day (POD) 365. Medication administration records were linked to procedures and transformed into an agent-day dataset with POD assignment. Organ-specific protocols defined a prophylaxis-window end day; post-prophylaxis analyses included antibiotic-days when POD exceeded end day. Broad-spectrum categories included Ped_SAAR_BSHO plus HighestRisk [Ped_SAAR_XDR (extensively drug-resistant) plus carbapenems]. SSI was chart-reviewed. A hierarchy assigned each post-prophylaxis broad-spectrum agent-day a primary context (culture, SSI, subsequent procedure, or residual unclassified). Antibiotic episodes (courses) used a locked gap rule (split if exposed-day gap ?3 days) and were linked to culture-days, SSIs or subsequent procedures to classify episode outcomes. **Results:** Over POD 0–365, patients contributed 10,064 hospital-days and received 9,774 antibacterial DOT (971.2/1,000 hospital-days); Ped_BSHO contributed 2,529 DOT (251.3/1,000 hospital-days). Ped_BSHO rates varied by organ (DOT/1,000 hospital-days: 76.3 heart, 273.0 liver, 105.4 kidney, 355.5 intestinal, 494.2 multivisceral). Following completion of index procedure prophylaxis, broad-spectrum exposure totaled 2,379 DOT-days; HighestRisk accounted for 667 DOT-days (435 multivisceral). Primary attribution classified post-prophylaxis broad-spectrum DOT-days as unclassified (982; 41.3%) or blood-culture rule-out (979; 41.1%); SSI treatment windows contributed 215 DOT-days (9.0%), and subsequent procedures contributed 66 DOT-days (2.8%). Unclassified DOT was concentrated (top 10 patients 64.8%) and occurred largely in intensive care units; piperacillin–tazobactam and meropenem predominated, and 24.5% of unclassified DOT-days were HighestRisk. From 1,390 blood culture accessions we defined 1119 sets, of which 94.7% (n=1060) were no growth, 3.4% (n=38) positive and 0.5% (n=6) contaminant. In the episode-based analysis, antibiotic courses that overlapped a blood-culture day were usually rule-out rather than culture-confirmed, and rule-out episodes contributed substantially more antibiotic burden than culture-positive episodes (DOT 1,510 vs 248). Culture intensity was high in multivisceral/intestinal PICU patients and PCICU heart patients. **Conclusions:** After prophylaxis ends, broad-spectrum exposure is dominated by evaluation-driven care and a large residual unclassified burden, while SSI-associated exposure remains a smaller, safety-anchored signal. This procedure-anchored, episode-based framework quantifies organ-specific burden and prioritizes targets for paired diagnostic and antibiotic stewardship.

**Figure:**
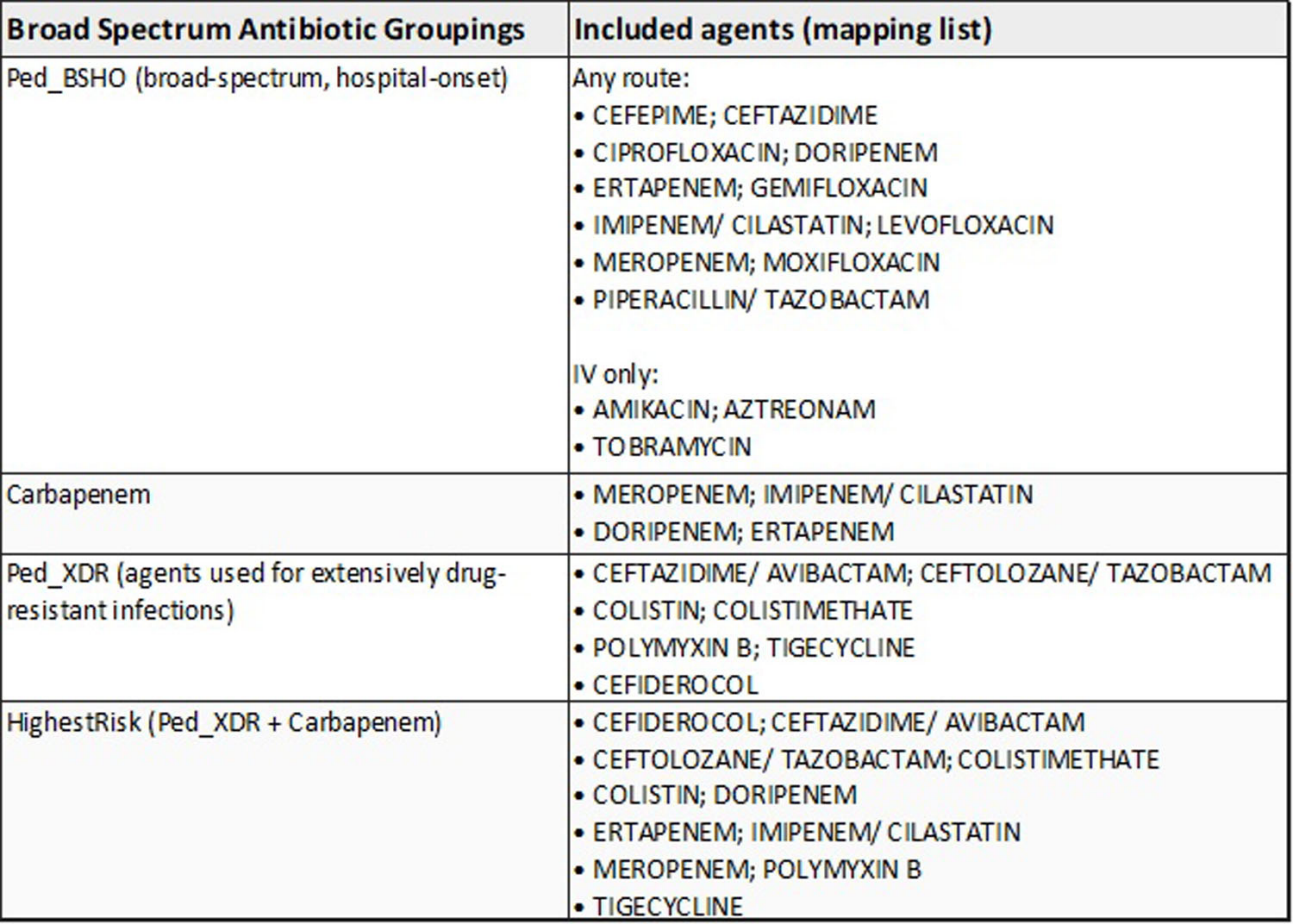

**Figure:**
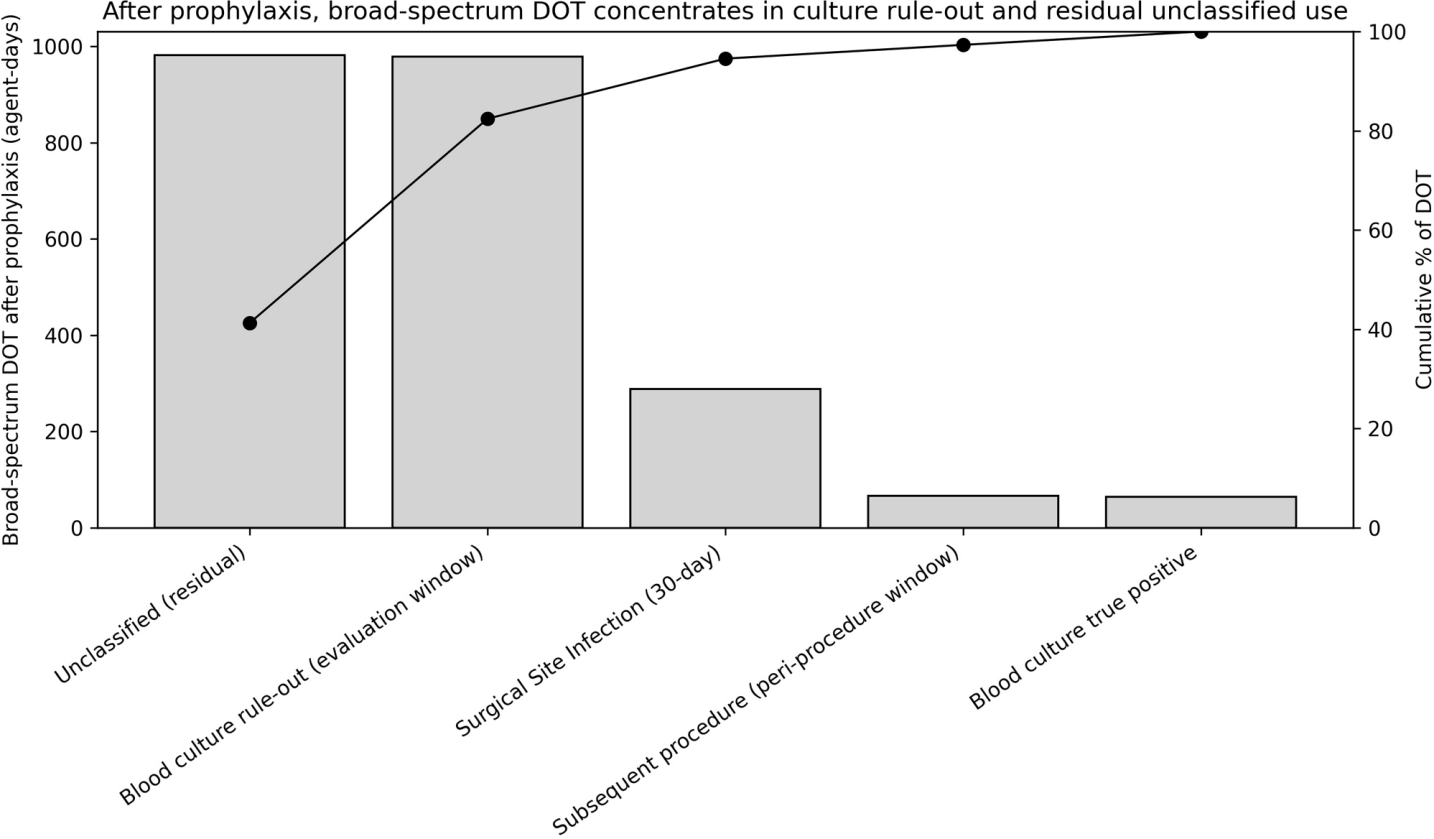

**Figure:**
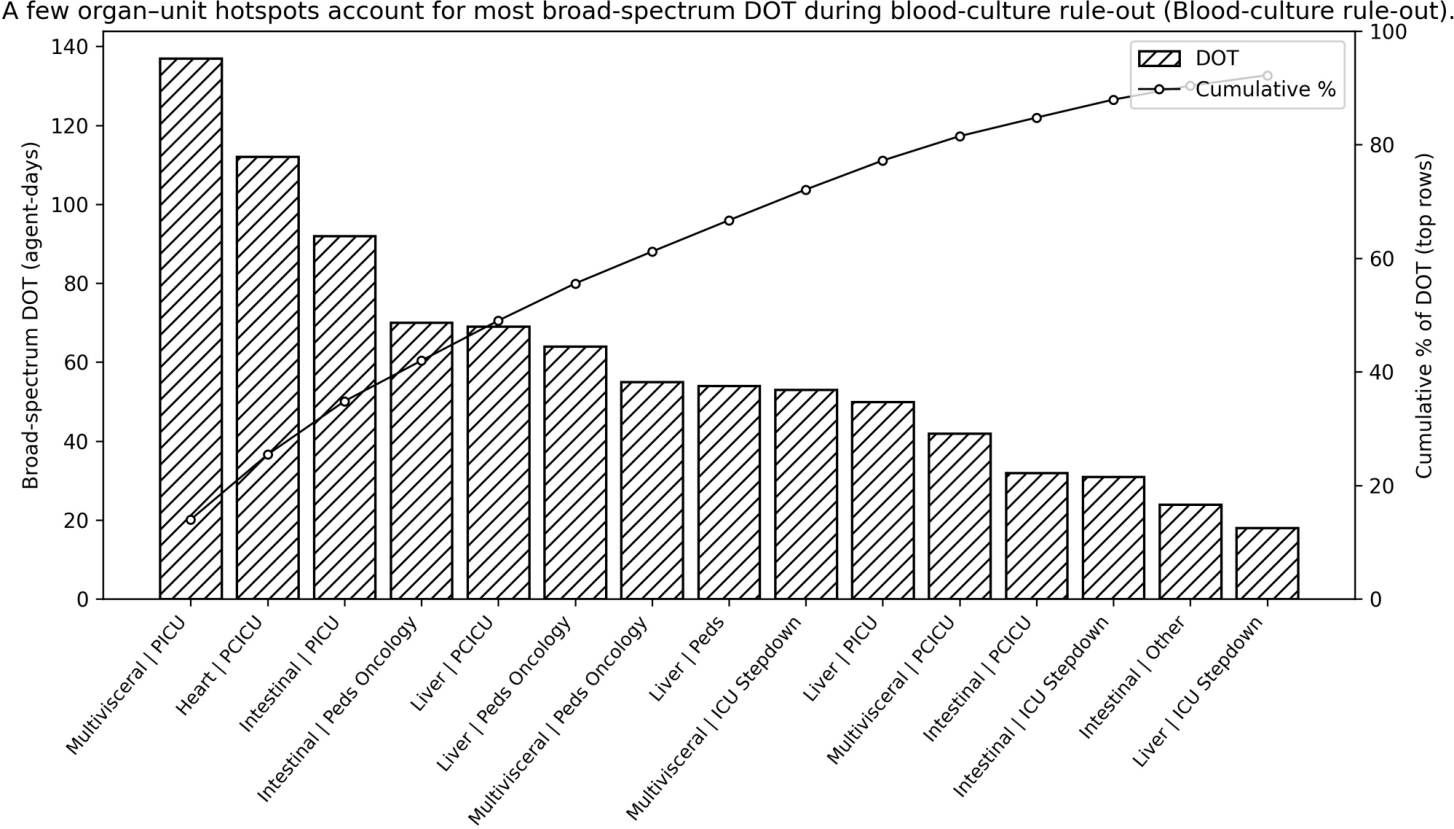

**Figure:**
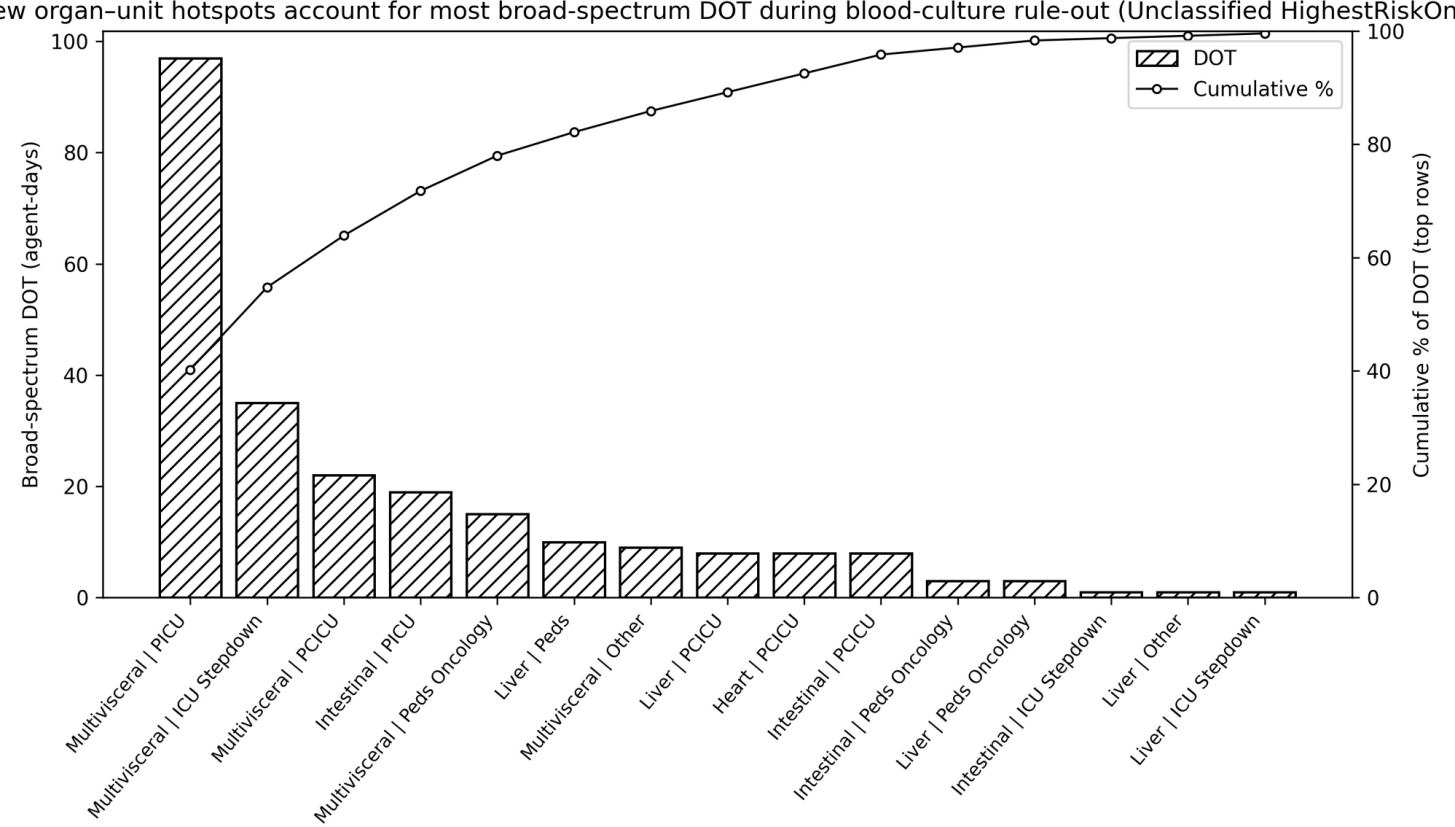

**Figure:**
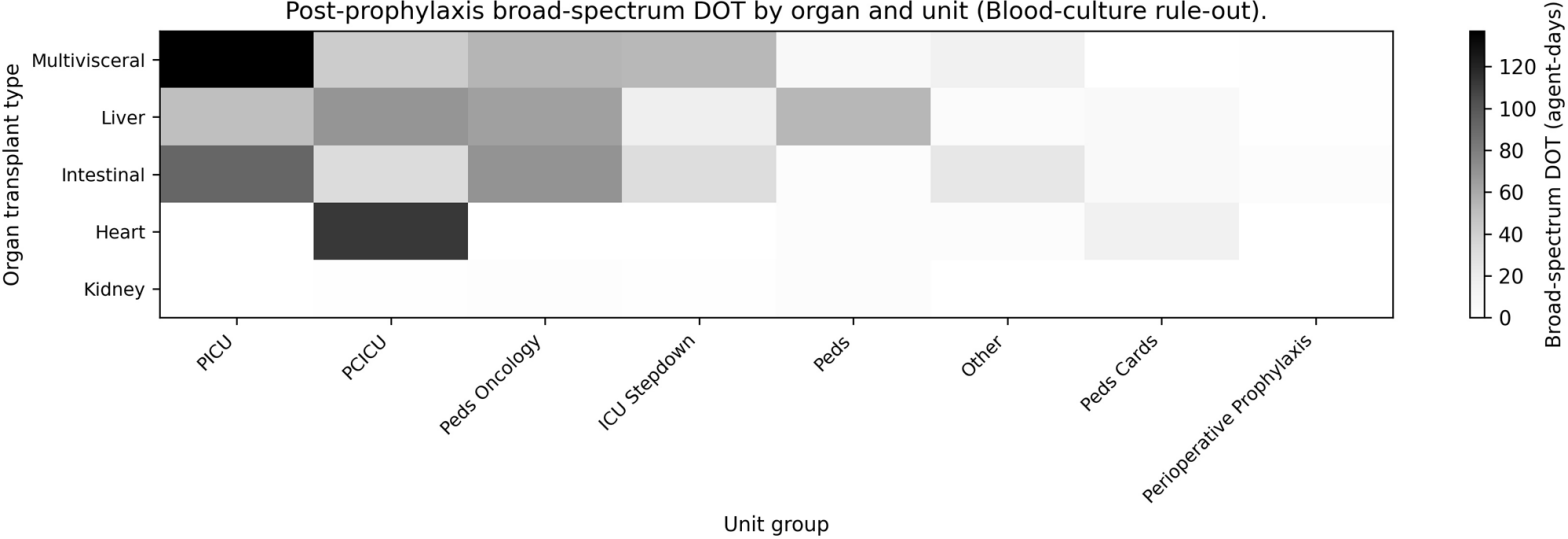

**Figure:**
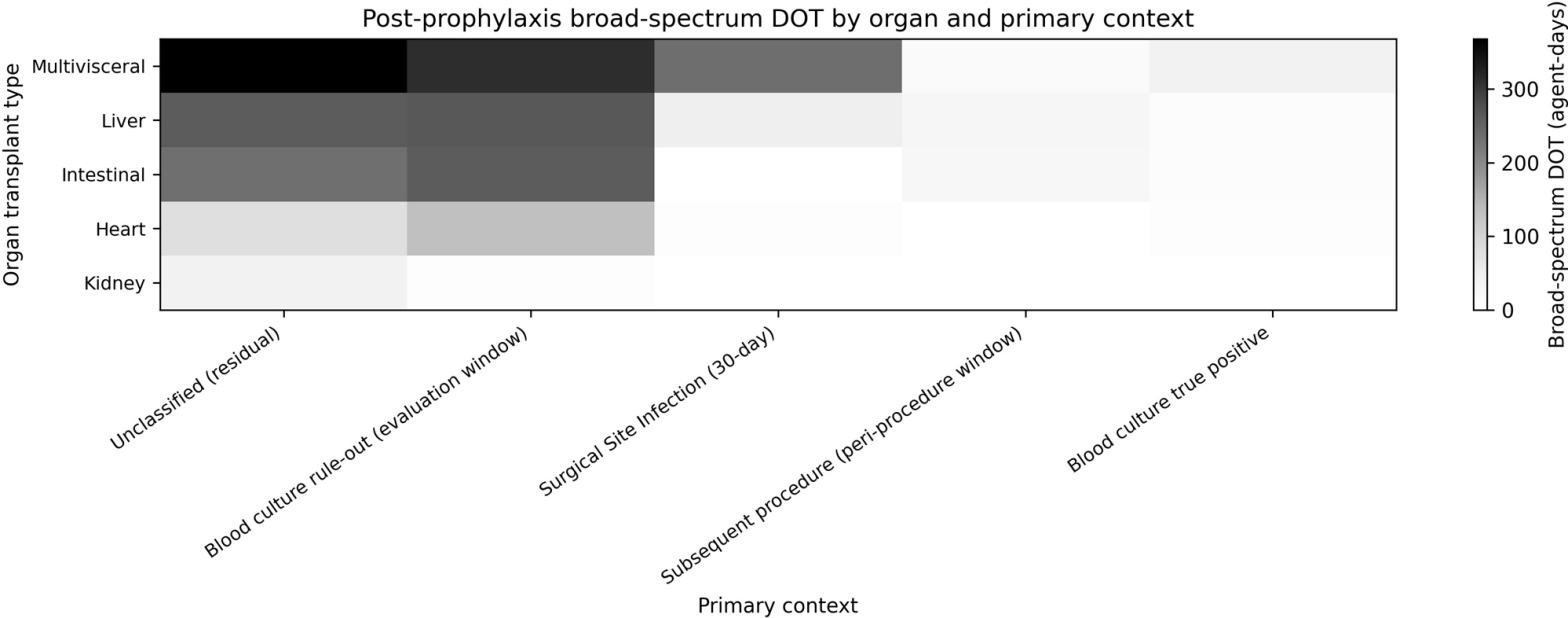

**Figure:**